# A new species of redfin (Teleostei, Cyprinidae, *Pseudobarbus*) from the Verlorenvlei River system, South Africa

**DOI:** 10.3897/zookeys.453.8072

**Published:** 2014-11-11

**Authors:** Albert Chakona, Ernst R. Swartz, Paul H. Skelton

**Affiliations:** 1 South African Institute for Aquatic Biodiversity, Private Bag 1015, Grahamstown 6140, South Africa

**Keywords:** Freshwater fish, endemic hotspot, threatened, double barbeled redfins

## Abstract

*Pseudobarbus verloreni*, a new species, is described from material collected in the Verlorenvlei River system on the west coast of South Africa. It differs from its congeners (except *Pseudobarbus skeltoni*, *Pseudobarbus burchelli*, and *Pseudobarbus burgi*) by the presence of two pairs of oral barbels. *Pseudobarbus verloreni* **sp. n.** can be distinguished from the three currently described double barbeled *Pseudobarbus* species by the following combination of characters: pigment pattern, generally deeper body relative to standard length, a longer intestine associated with the deeper body form, shorter snout relative to head length, and much shorter anterior barbels relative to head length. The new species is distinguished from *Pseudobarbus burgi* in the neighbouring Berg River system by its longer head and longer pre-dorsal length. It seems as if *Pseudobarbus verloreni* **sp. n.** has been extirpated from the Langvlei River system and face several threats to its survival in the Verlorenvlei River system.

## Introduction

Species of the cyprinid genus *Pseudobarbus* (commonly referred to as “redfins”) are distinctly pigmented small-to-medium sized riverine fishes endemic to southern Africa (Skelton 1988). *Pseudobarbus* was described by Smith (1841) as a subgenus of *Barbus*. Nearly 150 years later, the subgenus was raised to a full generic status (Skelton 1988). The monophyly of *Pseudobarbus* is supported by molecular data and morphological characters (Swartz et al. 2009). The most prominent diagnostic characters for members of this tetraploid genus (Naran et al. 2006) are the presence of bright red fins, a soft or flexible primary dorsal spine, and males develop prominent head and body tubercles during the breeding season. During his taxonomic revision of *Pseudobarbus*, Skelton (1988) recognised seven species, namely *Pseudobarbus afer* (Peters, 1864), *Pseudobarbus asper* (Boulenger, 1911), *Pseudobarbus burchelli* (Smith, 1841), *Pseudobarbus phlegethon* (Barnard, 1938), *Pseudobarbus quathlambae* (Barnard, 1938), and *Pseudobarbus tenuis* (Barnard, 1938). Two decades later, following genetic studies and a resurgence of field surveys in the Cape Floristic Region, a new redfin species *Pseudobarbus skeltoni* was described from the Breede River system (Chakona and Swartz 2013), bringing the number of nominal species to eight. Based on insights from molecular studies, several other taxa of this genus remain to be described (Bloomer and Impson 2000, Swartz et al. 2007, 2009, 2014, Chakona et al. 2013). Herein, we describe a ninth species of *Pseudobarbus* from the Verlorenvlei River system, which was identified as a unique genetic lineage of *Pseudobarbus burgi* following a phylogeographic study by Bloomer and Impson (2000).

The Verlorenvlei and Berg lineages of *Pseudobarbus burgi*, three lineages of *Pseudobarbus burchelli* (see Chakona et al. 2013 and Swartz et al. 2014), and *Pseudobarbus skeltoni* have two pairs of oral barbels and form a monophyletic group within *Pseudobarbus* (Swartz et al. 2009). The taxonomic history of the double barbeled redfins has been complex and confusing. The first double barbeled redfin was described by Smith (1841) as Barbus (Pseudobarbus) burchelli. This was followed by descriptions of *Gnathendalia vulnerata* Castelnau, 1861 and *Barbus multimaculatus* Steindachner, 1870, both from the Breede River system. Valenciennes (Cuvier and Valenciennes 1842) described *Barbus gobionides* but this species was later synonymised with *Gnathendalia vulnerata* by Günther (1868). Barnard (1943) subsequently declared *Barbus gobionides* Valenciennes, 1842 a nomen dubium, while Boulenger (1905) placed *Barbus multimaculatus* Steindachner, 1870 in synonymy with *Gnathendalia vulnerata*. This decision was subsequently accepted by Barnard (1943), Jubb (1965), and Skelton (1988). Boulenger (1911) described Barbus (Pseudobarbus) burgi from the Berg River system, but Barnard (1943) placed this species in synonymy with Barbus (Pseudobarbus) burchelli, and recognised *Barbus vulneratus* for the Breede River system. Jubb (1965) later reversed this decision and considered *Gnathendalia vulnerata* to be a synonym of Barbus (Pseudobarbus) burchelli. Skelton (1988) accepted Jubb’s (1965) nomenclatural changes to maintain taxonomic stability in his taxonomic revision of redfin minnows.

Thus, *Pseudobarbus burchelli*, *Pseudobarbus burgi*, and the recently described *Pseudobarbus skeltoni* are the only double barbeled *Pseudobarbus* species that are presently recognised. The known distribution of *Pseudobarbus burchelli* sensu lato spans four river systems (Heuningnes, Breede, Duiwenhoks, and Goukou) on the south coast of South Africa (Skelton 1988), while *Pseudobarbus skeltoni* is restricted to the Breede River system (Chakona and Swartz 2013). The historical distribution of *Pseudobarbus burgi* sensu lato included the Langvlei, Verlorenvlei, Berg, and Eerste river systems on the west coast of South Africa (Skelton 1988). The Eerste population is thought to be extinct due to a combination of impacts including introduction of non-native species (Gaigher et al. 1980). Recent surveys suggest that the Langvlei population has also been lost. Skelton (1988) noted that specimens of *Pseudobarbus burgi* from the Verlorenvlei River system had a longer intestine and longer predorsal length compared to specimens from the Berg River system. Bloomer and Impson (2000) discovered high levels of genetic differentiation (5.3–7.0% for the mitochondrial control region) between populations of *Pseudobarbus burgi* from the Verlorenvlei and Berg river systems, indicating a long history of isolation. The differentiation between these two lineages was confirmed by Swartz et al. (2009) and Chakona and Swartz (2013). The purpose of the present study is to describe the Verlorenvlei *Pseudobarbus* population as a new species, *Pseudobarbus verloreni* sp. n.

## Materials and methods

Institutional abbreviations follow Sabaj (2013) and are listed at http://www.asih.org/node/204. Description of the new *Pseudobarbus* species is based on 47 specimens (holotype and paratypes) that were collected from the Verlorenvlei River system during surveys conducted in January 1999 and March 2012. The type material has been deposited at the South African Institute for Aquatic Biodiversity (SAIAB), MRAC, USNM and BMNH.

### Molecular data

Two *Pseudobarbus* individuals from the Verlorenvlei River system were sequenced to assign a hologenetype and a paragenetype following Chakrabarty (2010) for the mitochondrial cytochrome *b* gene. The sequences were added to the genetic analysis done by Chakona and Swartz (2013) to show the phylogenetic position of the hologenetype and the paragenetype in relation to all known lineages and species of *Pseudobarbus*. Methods of DNA extraction, amplification, sequencing and analysis follow Swartz et al. (2009) and Chakona and Swartz (2013). The hologenetype and paragenetype sequences were deposited in GenBank for future reference (GenBank numbers are given below) following the definitions of Chakrabarty (2010).

### Morphological data

Meristic and morphological characters were examined following Hubbs and Lagler (1958), Skelton (1988), and Chakona and Swartz (2013). The characters considered for each specimen in the present study (22 morphometric measurements) and (12 meristic counts) are presented in Table 1. In addition, entire branchial baskets were dissected from three specimens to examine and count pharyngeal teeth.

**Table 1.** Morphological characters of *Pseudobarbus* species used in the present study.

Character	Description	Acronym
*Morphometric measurements*		
Standard length	Tip of the snout to the point of flecture of the caudal fin	SL
Pre-dorsal length	Tip of the snout to the origin of the dorsal fin	PDL
Head length	Tip of the snout to the posterior bony margin of the operculum	HL
Snout length	Tip of the snout to the anterior bony edge of the orbit	S
Orbit diameter	The greatest bony diameter of the orbit	OD
Inter-orbit width	Shortest distance between bony edges of the orbits	IO
Post-orbit length	Distance between the posterior bony edge of orbit to the posterior bony edge of operculum	PO
Head depth	Maximum depth measured from the nape	HD
Body depth	Maximum depth measured from the anterior base of the dorsal fin	BD
Anterior barbel length	From base to tip of anterior barbel	AB
Posterior barbel length	From base to tip of posterior barbel	PB
Dorsal fin base	Distance between origin of dorsal fin and base of last dorsal fin ray	DB
Dorsal fin height	From anterior base to tip of dorsal fin	DH
Pectoral fin length	From anterior base to tip of pectoral fin	PtL
Pelvic fin length	From anterior base to tip of pelvic fin	PvL
Anal fin base	Distance between origin of anal fin and base of last anal fin	AfB
Anal fin height	From anterior base to tip of anal fin	AfH
Caudal peduncle length	Distance from posterior base of anal fin the point of flecture of the caudal fin	CPL
Caudal peduncle depth	The least depth of the caudal peduncle	CPD
Pectoral to pelvic fin length	Distance between the posterior margins of the fin bases	PP
Pelvic to anal fin length	Distance between posterior base of the pelvic fin to anterior base of the anal fin	PA
Body width	The greatest width just anterior to the origin of the dorsal fin	BW
*Meristic counts*		
Lateral line scales	Number of scale rows along the lateral line	LL
Lateral line to dorsal fin origin	Number of scale rows between lateral line scale row (does not include lateral line scale) and anterior base of the dorsal fin	LD
Lateral line to pelvic fin origin	Number of scale rows between lateral line scale row (does not include lateral line scale) and anterior base of pelvic fin	LP
Lateral line to anal fin origin	Number of scale rows between lateral line scale row (does not include lateral line scale) and anterior base of the anal fin	LA
Circumpeduncular scales	Number of scale rows around the caudal peduncle at narrowest portion of caudal peduncle	CP
Predorsal scales	Number of scale rows between the supraoccipital and the anterior base (origin) of the dorsal fin	PDS
Unbranched dorsal fin rays	Number of unbranched primary dorsal rays	UdR
Branched dorsal fin rays	Number of branched dorsal rays; two last branched rays counted as one	BdR
Anal fin rays	Includes both simple and branched rays; two last rays counted as one	
Pectoral fin rays	Includes both simple and branched rays	
Pelvic fin rays	Includes both simple and branched rays	
Total vertebrae	Total number of vertebrae in vertebral column (including four Weberian vertebrae and a single ural centrum)	TV
Pre-dorsal vertebrae	Total number of vertebrae in advance of the leading dorsal fin pterigiophore (including the four Weberian vertebrae)	PdV
Pre-caudal vertebrae	Total number of vertebrae in advance of the vertebrae with haemal arch opposite the leading anal pterygiophore plus the four Weberian vertebrae	PcV
Pre-anal vertebrae	Total number of vertebrae in advance of the leading anal pterygiophore (including the four Weberian vertebrae)	PaV
Caudal vertebrae	Total number of vertebrae posterior to (and including) the vertebra with haemal arch opposite the leading anal pterygiophore plus a single ural centrum	CV

We compared morphological and meristic differences among all double barbeled redfins using raw data from Skelton (1980, 1988) and Chakona and Swartz (2013). Specimens were assigned to four groups based on geographic origin and previous genetic results (Bloomer and Impson 2000; Swartz et al. 2009, 2014; Chakona and Swartz 2013; Chakona et al. 2013): *Pseudobarbus skeltoni* (*n*=25), *Pseudobarbus burchelli* (*n*=128), *Pseudobarbus burgi* (specimens from the Berg River system only; *n*=66) and Verlorenvlei *Pseudobarbus* (specimens from the Verlorenvlei River system only; *n*=47). A total of 47 specimens of Verlorenvlei *Pseudobarbus* were radiographed to count skeletal features.

Statistical analyses were performed with the programs InfoStat (Di Rienzo et al. 2012), PAST and STATISTICA 12. Prior to analyses, morphometric data were normalised using procedures described by Lleonart et al. (2000). Analyses of meristic characters were performed using the raw data.

Principal component analysis (PCA) was performed using the correlation matrix to explore the separation of the specimens based on the normalised morphometric data (Lleonart et al. 2000) and raw meristic characters. Invariant characters (such as the number of pelvic fin rays) were excluded from analysis. All scores (including PC1) were considered, because the normalisation approach allows for size free comparisons (Lleonart et al. 2000).

Discriminant Function Analysis (DFA) was performed to visualise the degree of morphological separation among the species and to identify the most important characters that contribute to the differentiation. DFA also provides jacknifed measurements of re-classification success of individuals to their original group, as well as identifying the group to which individuals were assigned if misclassified. Separate DFAs were performed for the morphometric and meristic characters, as well as for these two data sets combined.

## Results

**Figure 1.**
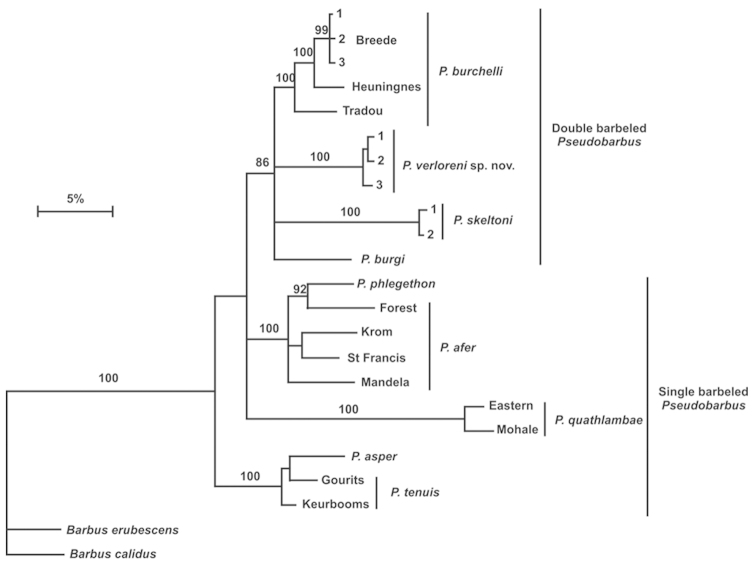
Bayesian phylogenetic tree showing genetic distances between *Pseudobarbus verloreni* sp. n. compared to all other *Pseudobarbus* species/lineages. Bayesian posterior probabilities are shown on the branches.

Figure 1 is a re-analysis of the phylogeny done by Chakona and Swartz (2013) to include the genetypes of *Pseudobarbus verloreni* sp. n. sequenced in the present study. It shows the phylogenetic relationships among double barbeled redfins based on the mitochondrial cytochrome *b* data, and shows the position of the new species that is distinct from the three described double barbeled redfin species, including the three previously identified lineages of *Pseudobarbus burchelli* (Swartz et al. 2009, 2014). The model corrected genetic distances show deep divergences (6.6–12.3%) between the new species and the other members of the double barbeled redfin group.

**Figure 2.**
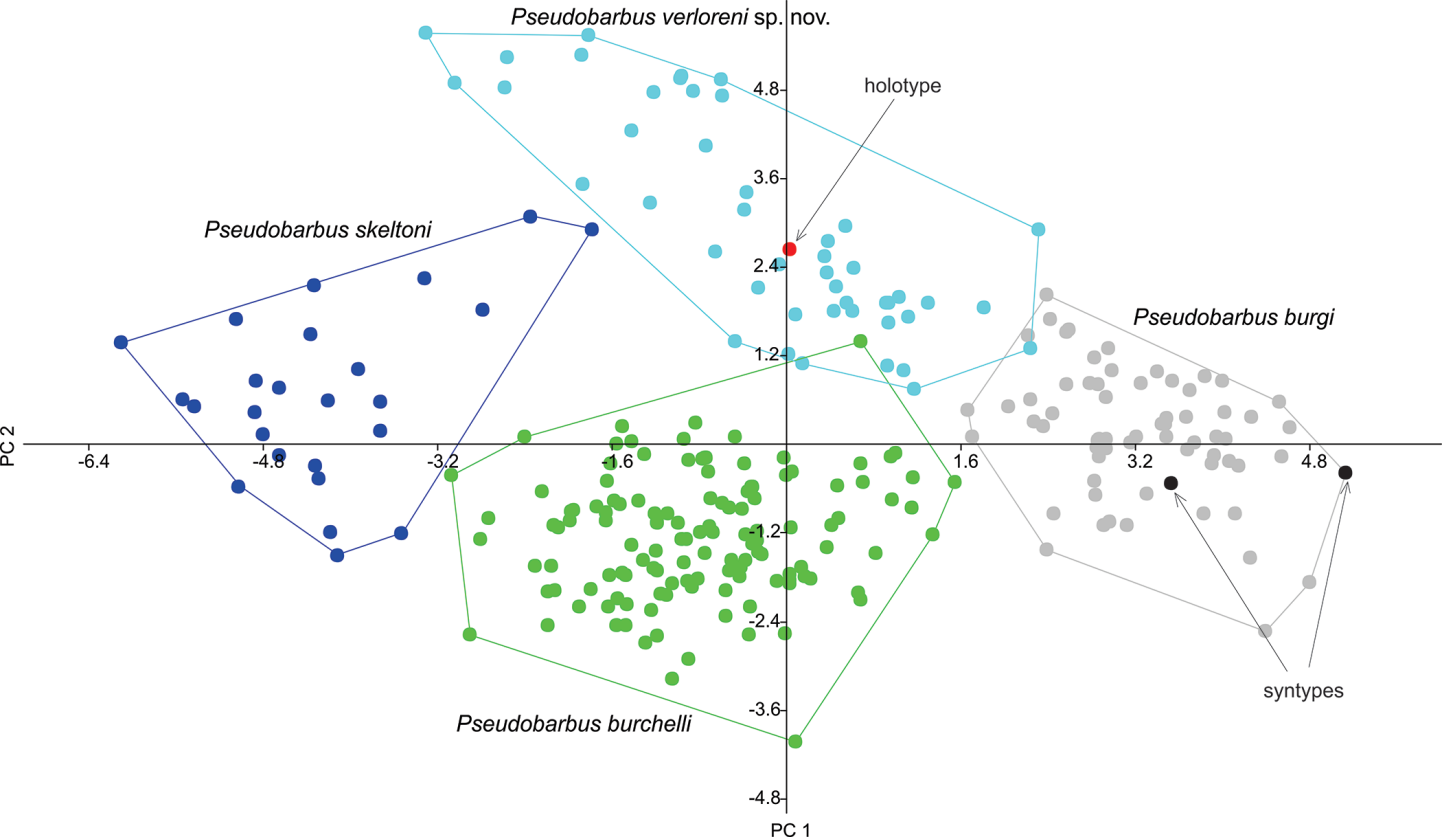
Scatter plot of PC1 against PC2 for a PCA carried out on 15 normalised morphometric and seven raw meristic characters for all examined specimens (*n*=266) of double barbeled redfins from the Cape Floristic Region of South Africa. The figure shows clear separation of *Pseudobarbus verloreni* sp. n. from to all the other *Pseudobarbus* species/lineages.

Principal components analysis (PCA) of normalised morphometric and raw meristic characters shows *Pseudobarbus* specimens from the Verlorenvlei River system, herein described as the new species *Pseudobarbus verloreni* sp. n., form a cluster that is clearly separated from *Pseudobarbus skeltoni* and marginally overlaps with *Pseudobarbus burchelli* and *Pseudobarbus burgi* (Figure 2). The most important factor loadings are presented in Table 2. PCI was mainly defined by differences in head length, head depth, predorsal length, number of lateral line scales, number of scale rows between lateral line and dorsal fin, number of scale rows around the caudal peduncle and the number of predorsal scale rows (Table 2). PCII primarily contrasted differences in body depth, length of anterior barbel, and snout length. PCIII was mainly defined by caudal peduncle depth and body width. Specimens of *Pseudobarbus verloreni* sp. n. were associated positively with PCII, describing individuals characterised by deeper bodies relative to standard length. Specimens of the new species are separated from those of *Pseudobarbus burgi*, which were associated positively with PCI, describing individuals characterised by deeper heads relative to head length. Note also that the syntypes of *Pseudobarbus burgi* are clearly not conspecific with specimens of *Pseudobarbus verloreni* (Figure 2).

**Table 2.** Factor loadings for the first three principal component (PC) axes of a PCA carried out on morphometric and meristic characters of double barbeled *Pseudobarbus* specimens (*n*=266) from the Cape Floristic Region of South Africa.

Character	PCI	PCII	PCIII
Head length	-0.769	0.408	-0.077
Head depth	0.669	0.194	-0.276
Inter orbit	0.514	0.297	-0.421
Snout length	0.205	-0.727	0.040
Post orbit	0.325	-0.387	-0.067
Predorsal length	-0.721	0.418	0.218
Dorsal fin base	0.507	-0.115	0.296
Body depth	0.148	0.796	0.269
Body width	-0.463	0.153	0.620
Caudal peduncle length	0.478	-0.431	0.107
Caudal peduncle depth	-0.335	0.178	0.752
Anterior barbel	-0.405	-0.729	0.418
Posterior barbel	-0.250	-0.477	0.520
Unbranched dorsal fin rays	-0.215	-0.477	0.111
Lateral line scales	-0.650	-0.340	-0.153
Scale rows between lateral line and dorsal fin	-0.853	0.024	-0.100
Scale rows between lateral line and pelvic fin	-0.575	0.248	-0.191
Scale rows between lateral line and anal fin	-0.180	0.165	0.114
Scale rows around caudal peduncle	-0.687	-0.103	-0.194
Predorsal scale rows	-0.758	-0.352	0.095

The Discriminant Function Analysis (DFA) performed using combined morphometric and meristic characters correctly classified all individuals of the new species (Table 3). Similar to PCA results, the discriminant scores showed that the new species was clearly separated from the three previously described species of *Pseudobarbus* with two pairs of oral barbels (results not shown).

**Table 3.** Classification results of discriminant function analysis using (a) combined morphometric and meristic characters, (b) morphometric characters and (c) meristic characters of double barbeled *Pseudobarbus* species from the Cape Floristic Region of South Africa.

	Species	Predicted count				Total	Error (%)
		1	2	3	4		
*Combined data*	1. *Pseudobarbus burchelli*	128	0	0	0	128	0.00
	2. *Pseudobarbus burgi*	0	66	0	0	66	0.00
	3. *Pseudobarbus skeltoni*	0	0	24	1	25	4.00
	4. *Pseudobarbus verloreni* sp. n.	0	0	0	47	47	0.00
*Morphometrics*	1. *Pseudobarbus burchelli*	124	1	3	0	128	3.13
	2. *Pseudobarbus burgi*	0	66	0	0	66	0.00
	3. *Pseudobarbus skeltoni*	1	0	22	2	25	12.00
	4. *Pseudobarbus verloreni* sp. n.	0	0	0	47	47	0.00
*Meristics*	1. *Pseudobarbus burchelli*	99	9	5	15	128	22.66
	2. *Pseudobarbus burgi*	0	65	0	1	66	1.52
	3. *Pseudobarbus skeltoni*	0	0	25	0	25	0.00
	4. *Pseudobarbus verloreni* sp. n.	1	2	0	44	47	12.41

The DFA using morphometric measurements revealed morphological shape differences between the new species and the other previously described species of *Pseudobarbus* with two pairs of oral barbels. This analysis correctly classified all individuals of the new species, *Pseudobarbus verloreni* sp. n., as well as *Pseudobarbus burgi* sensu stricto while three individuals of *Pseudobarbus skeltoni* and four individuals of *Pseudobarbus burchelli* were misclassified (Table 3).

In contrast, the DFA using meristic characters showed poor classification of individuals of the four species, with three individuals of the new species, 29 individuals of *Pseudobarbus burchelli* and one individual of *Pseudobarbus burgi* being misclassified (Table 3).

Based on the deep genetic and significant morphological divergence between individuals from the Verlorenvlei River system and other members of the double barbeled redfin group, the Verlorenvlei population represents a new species.

### Pseudobarbus verloreni sp. n.

Taxon classificationAnimaliaCypriniformesCyprinidae

http://zoobank.org/A98AABCD-73D2-425B-877A-22C7364A57B3

Figure 3 , Table 4

#### Proposed common names.

Verlorenvlei redfin (English), Verlorenvlei rooivlerkie (Afrikaans).

#### Holotype.

South Africa: Western Cape Province: SAIAB186092, mature male, 70.8 mm standard length (SL), collected from the Verlorenvlei River, 20 m upstream from railway at the Het Kruis bridge on R365 (32.60179000 S, 18.75039000 E) on 13 March 2012 by E. Swartz and W. Bronaugh, using a seine net. Hologenetype: GenBank number KM366106.

#### Paratypes

(*n*=46). South Africa: Western Cape Province: SAIAB192542 (*n*=3, 53.3–70.8 mm SL), same data as for holotype; SAIAB59808 (*n*=10, 40.1–46.9 mm SL), collected from the Verlorenvlei River (32.74560165 S, 18.81780052 E) on 22 January 1999 by R. Bills and D. Naran using a seine net and D-net; BMNH2014.2.26.1-2 (*n*=2, 52.8–58.2 mm SL), USNM427302 (*n*=2, 53.4–56.8 mm SL), MRAC-B4-03-P-1-2 (*n*=2, 53.0–54.5 mm SL), same data and collectors as SAIAB59808, SAIAB121038 (*n*=10, 34.0–68.0 mm SL) collected from the Verlorenvlei River in 1973 by P. Skelton, C. Gaigher and D. Heard; SAIAB121039 (*n*=17, 41–57 mm SL) collected from the Kruis River, Verlorenvlei, in 1973 by P. Skelton, C. Gaigher and D. Heard. Paragenetype: SAIAB192542, GenBank number: KM366107.

#### Diagnosis.

The new species can be distinguished from its congeners by distinct linear speckles above and below the lateral line, anterior barbels minute and much smaller than eye diameter, lips unretracted, and a cartilaginous fig absent.

#### Description.

Proportional measurements and meristic characters are presented in Table 4. The body is fusiform, more or less laterally compressed, with a conspicuous lateral stripe from the posterior edge of the head terminating in a dark spot at the base of the caudal peduncle. The lateral band is more pronounced in juveniles and sub-adults, but is less conspicuous in adults. Distinct linear speckles are present on the abdomen. The head is relatively small and slightly depressed; head length is almost equal to body depth. Two pairs of barbels, rostral (anterior) barbels minute and much smaller than eye diameter; maxillary barbels are rooted at the corner of the mouth are longer than rostral barbels and are equal or smaller than eye diameter. Eyes are relatively large, located dorsolaterally, closer to the tip of the snout than the caudal margin of the operculum, interorbital space is flat. Mouth is sub-terminal, lower lip is unretracted and lacks a cartilaginous fig. Snout is relatively short, only few nuptial tubercles present (observed in only one individual; Figure 4) or tubercles are completely lacking.

**Table 4.** Comparisons of the morphometric measurements and meristic counts of *Pseudobarbus* species with two pairs of barbels. For meristics, the mode is given first, with the range in parentheses. Values are expressed as mean ± SE.

	*Pseudobarbus verloreni* sp. n.		*Pseudobarbus burgi*	*Pseudobarbus skeltoni*	*Pseudobarbus burchelli*
	Holotype	Paratypes			
No. of specimens	1	46	66	25	128
Standard length (SL) (mm)	70.0	34.0–70.8	42.0–109.0	28.5–163.4	30.0–151.7
Head length (HL) (mm)	18.9	10.0–19.6	10.9–25.0	8.6–51.7	8.1–45.8
Percentage of SL (%)					
Head length	27.0	28.1±0.2	24.9±0.1	30.5±1.5	26.8± 1.0
Predorsal length	54.0	53.1±0.4	47.6±0.1	53.3± 1.7	51.2± 1.4
Dorsal fin base	12.9	13.2±0.1	13.7±0.1	12.0± 0.8	13.4± 0.7
Dorsal fin height	25.3	25.6±0.3	24.2±0.1	21.2± 1.8	22.9± 1.4
Body depth	27.6	29.1±0.2	26.9±0.3	25.6± 1.3	25.6± 1.6
Body width	16.1	16.4±0.2	15.2±0.3	17.9± 1.5	17.1± 1.6
Caudal peduncle length	20.9	23.5±0.2	25.7±0.1	22.4± 0.8	25.0± 1.2
Percentage of HL (%)					
Head depth	72.5	73.0±0.4	74.1± 0.3	64.2± 3.1	70.1± 2.6
Inter-obit	36.0	34.4±0.3	33.1±0.3	28.1± 2.1	31.3± 2.1
Snout length	31.2	31.0±0.4	36.6±0.4	38.0± 2.2	36.5± 1.9
Post orbit	46.0	47.6±0.5	46.4±0.3	45.4± 1.8	45.2± 1.9
Anterior barbel length	6.3	3.3±0.3	5.1±0.2	20.3± 9.5	16.5± 4.3
Posterior barbel length	30.7	20.8±0.8	19.3±0.3	27.5± 11.9	28.4± 5.6
Orbit diameter	31.2	31.5±0.4	28.8±0.4	21.5± 4.4	27.7± 2.8
Percentage of caudal peduncle length (%)					
Caudal peduncle depth	61.0	52.6±0.8	46.9± 4.0	53.4± 3.6	49.4± 3.5
Unbranched dorsal fin rays	iv	iii (iii-v)	iii (iii–iv)	iii (iii–iv)	iv (iii–iv)
Branched dorsal fin rays	7	7 (7–8)	7 (6–7)	7 (7–8)	7 (6–8)
Unbranched anal fin rays	iii	iii (iii-iv)	iii (ii-iv)	iii (iii-iv)	iii (iii-iv)
Branched anal fin rays	5	5	5 (5–6)	5 (4–5)	5 (4–6)
Pectoral fin rays	14	15 (13–16)	14 (13–16)	13 (13–16)	14 (13–16)
Pelvic fin rays	8	8 (7–9)	8 (8–9)	8 (7–8)	8 (7–8)
Lateral line scales	33	32 (29–36)	32 (28–37)	38 (36–39)	35 (29–37)
Scale rows between lateral line and dorsal fin	6	6 (5–6)	5 (4–6)	7 (6–7)	6 (5–7)
Scale rows between lateral line and pelvic fin	4	5 (4–5)	4 (3–5)	5 (5–7)	4 (4–5)
Scale rows between lateral line and anal fin	4	4 (4–5)	4 (3–4)	5 (4–6)	4 (4–6)
Caudal peduncle scale rows	12	12 (12–16)	12 (12–13)	16 (15–18)	12 (12–16)
Predorsal scale rows	16	16 (13–18)	15 (12–16)	19 (17–21)	17 (14–22)
Total vertebrae	36	36 (34–37)	37 (35–38)	37 (36–38)	36 (35–37)
Pre-caudal vertebrae	19	19 (18–21)	19 (18–20)	20 (19–21)	19 (17–20)
Caudal vertebrae	17	17 (16–19)	18 (16–19)	17 (16–18)	18 (17–20)
Predorsal vertebrae	11	11 (10–13)	11 (10–12)	12	12 (11–13)
Pre-anal vertebrae	20	20 (19–21)	20 (19–22)	21 (20–22)	19 (18–21)

Counts for the holotype are given in a separate column in Table 4. Dorsal fin of the new species has 3 unbranched and 7 or 8 branched rays, distal margin almost straight, anterior base of dorsal fin inserted directly above or slightly in front of the origin of pelvic fins. Origin of dorsal fin inserted almost midway between tip of snout and base of caudal fin. Pectoral fins with 13 to 16 rays, shorter than head length, reaching beyond halfway to pelvic fin origin. Pelvic fin with 7 to 9 rays, shorter than head, posterior edge gently rounded, reaching the anus in males and within one or two scale rows to the anus in females. Anal fin with 3 or 4 unbranched and 5 branched rays, distal margin almost straight or gently rounded, origin inserted closer to origin of pelvic fin than base of caudal fin. Caudal fin forked, shorter than head length. Genital opening situated adjacent to anterior base of anal fin.

Scales moderately large; lateral line complete, majority of specimens have 32 scales along lateral line (range 29–36); 5–6 (mode 6) scale rows between dorsal fin origin and lateral line; 4–5 (mode 5) scale rows between pelvic fin origin and lateral line; 4–5 (mode 4) scale rows between lateral line and anal fin origin; 12–16 (mode 12) circumpeduncular scale rows. Predorsal scale rows 13–18 (mode 16), embedded in skin, smaller than flank scales. Patch between head and posterior base of pectoral fins naked; scales between posterior base of pectoral fins and anterior base of pelvic fins smaller than flank scales and embedded. Pelvic fins lack prominent or elongate axillary scales. Scales radiately striated.

Nuptial tubercles have only been observed in one individual of *Pseudobarbus verloreni* (Figure 4). The bilateral placement of tubercles on the snout is typical for *Pseudobarbus*, but the low number (only 4 tubercles in total) of the *Pseudobarbus verloreni* individual in Figure 4 is unusual. The other members of the double barbeled redfin group develop multiple prominent conical tubercles on the snout and head dorsum during the breeding season (see Chakona and Swartz 2013). Additional sampling during the breeding season is required to determine whether this is a consistent development pattern for *Pseudobarbus verloreni*.

#### Colouration.

**Figure 3.**
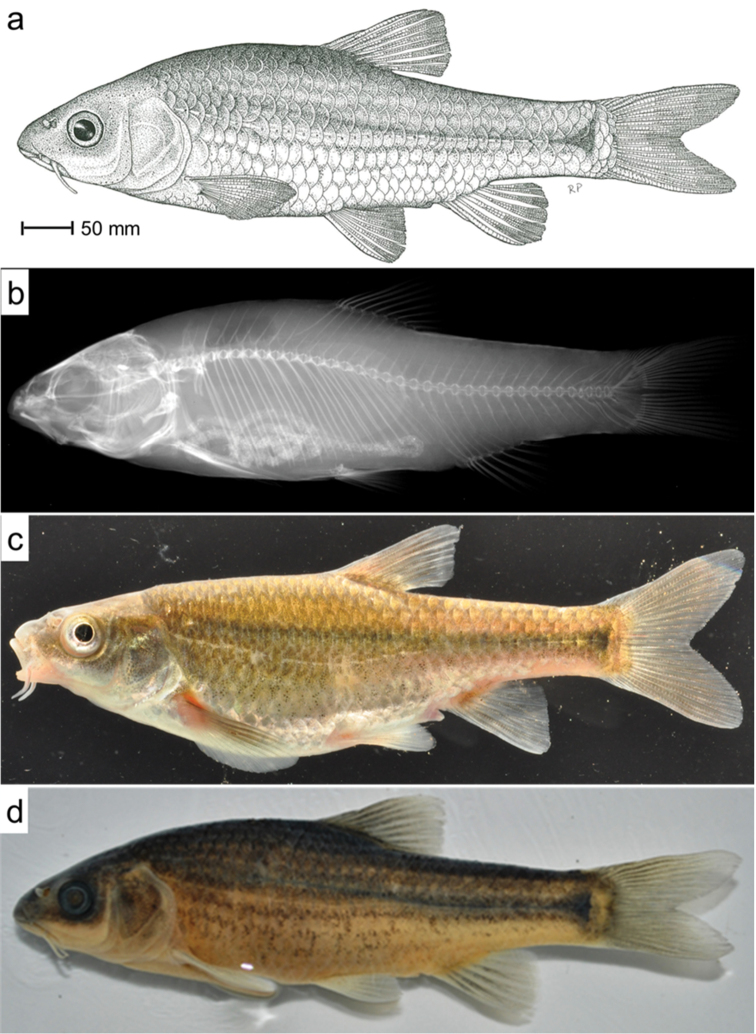
**a** Habitus of *Pseudobarbus verloreni* sp. n. (holotype, SAIAB186092). Drawing by R. Palmer **b** Radiograph of *Pseudobarbus verloreni* sp. n. (holotype, SAIAB186092) **c** Live colours of *Pseudobarbus verloreni* sp. n. (SAIAB186108). Picture by W. Bronaugh **d** Preserved colours of *Pseudobarbus verloreni* sp. n. (holotype, SAIAB186092).

Live colouration is golden-tan dorsally and laterally, becoming lighter and more silver ventrally (Figure 3c). In adults (above 40 mm SL), base of fins is pale red or yellowish-orange in some specimens. Alcohol preserved specimens have conspicuous linear speckles above and below the lateral line.

**Figure 4.**
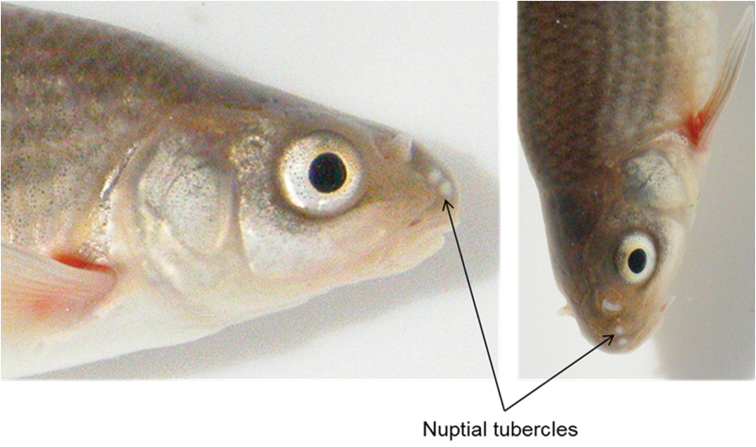
Lateral and dorsal view of *Pseudobarbus verloreni* sp. n. from site 4 in Figure 5. The figure shows a different pattern of tubercule expression compared to other members of the double barbeled redfin group (see Chakona and Swartz 2013).

#### Osteology.

Radiographs of the holotype (SAIAB 186092) and paratypes show that the species has osteology typical of all *Pseudobarbus* species. Compared to other cyprinids, particularly those within the genus *Barbus*, supraneural bones are less developed or extremely vestigial in all members of the genus *Pseudobarbus* (Skelton, 1988). Skelton (1988) did not record any supraneural bones in *Pseudobarbus* specimens (*n*=53) from the Verlorenvlei River system (herein described as *Pseudobarbus verloreni* sp. n.). Vertebrae counts for the holotype are given in a separate column in Table 4. Total number of vertebrae in 47- specimens investigated in the present study ranged from 34–37: 34 (*n*=1), 35 (*n*=3), 36 (*n*=31) or 37 (*n*=12) comprising 18–21 (mode 19) precaudal, 19–22 (mode 20) pre-anal, 10–13 (mode 11) predorsal and 16–19 (mode 17) caudal vertebrae (Table 4).

#### Additional information.

SAIAB59813, juveniles (n=68, 13.5–28.4 mm SL) and adults (n=3, 59.3–64.6 mm SL), collected from the Verlorenvlei River, near Grootfontein farm (32.39830017 S, 18.47419930 E) on 23 January 1999 by R. Bills and D. Naran using a seine net and D-net. Juveniles and sub-adults of *Pseudobarbus verloreni* have a conspicuous lateral band, while the lateral band is either less prominent or interrupted by linear spots in juveniles and sub-adults of the other double barbeled *Pseudobarbus* species. The new species has three rows of pharyngeal teeth, teeth pattern 2.3.5–5.3.2 (observed in 3 adults; SAIAB59813); teeth with asymmetrical crowns and hooked at their tips. *Pseudobarbus verloreni* sp. n. has the longest intestine relative to standard length compared to all the *Pseudobarbus* species (Skelton 1988: Figure 25Bc).

#### Comparisons.

*Pseudobarbus verloreni* sp. n. is distinguished from all other species of *Pseudobarbus* (except *Pseudobarbus skeltoni*, *Pseudobarbus burchelli* and *Pseudobarbus burgi*) by the presence of two pairs of oral barbels. The new species is distinguished from *Pseudobarbus skeltoni*, *Pseudobarbus burchelli* and *Pseudobarbus burgi* by having a deeper body relative to standard length, smaller anterior barbels and shorter snout relative to head length (Table 4). The new species is distinguished from *Pseudobarbus skeltoni* by having a sub-terminal mouth (versus terminal in adults of the latter species) and a smaller head relative to standard length (Table 4). *Pseudobarbus verloreni* is distinguished from *Pseudobarbus burchelli* and *Pseudobarbus skeltoni* by a deeper head, wider distance between the eyes (inter-orbit), larger eye relative to head length, shorter posterior barbel relative to head length, wider post-orbit distance, shallower caudal peduncle and generally fewer scales along the lateral line. *Pseudobarbus verloreni* is distinguished from *Pseudobarbus burchelli* and *Pseudobarbus burgi* by lack of cartilaginous fig on lower lip and having unretracted lips. The new species is distinguished from *Pseudobarbus burgi* by its longer head, longer pre-dorsal length, shorter caudal peduncle and larger eye (Table 4).

#### Reproduction.

Unknown, but spawning possibly occurs around October-December, based on the general patterns of congeners.

#### Distribution and habitat.

**Figure 5.**
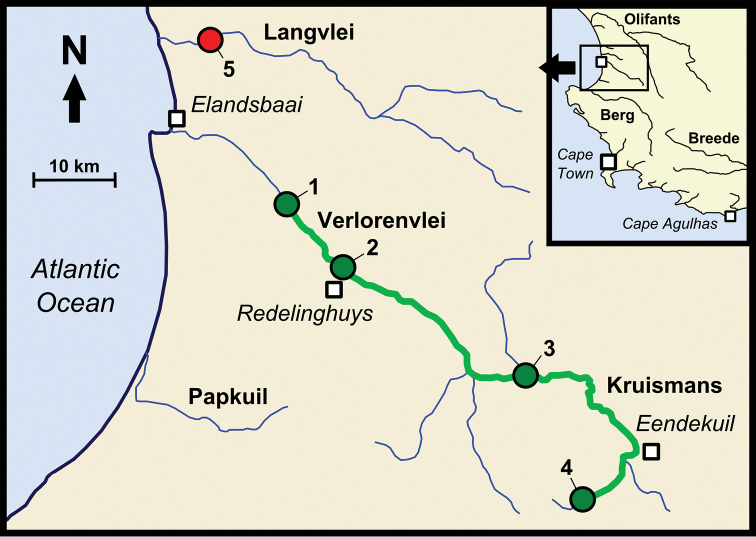
Map of a part of the west coast of South Africa. The map shows the likely present distribution of *Pseudobarbus verloreni* in the Verlorenvlei River system (green line), based on available accurate museum data (green circles; site 1=59813; site 2=SAIAB 59804, 130461 and 186108; site 3=SAIAB 121039, 128824, 186092 and 192542; site 4=SAIAB 130453 and 59808). Also shown is site 5 where the species was collected in the Langvlei River system in 1986 (red circle; site 5=SAIAB 130464), but have not been found in subsequent surveys. The insert map shows the study area in relation to Cape Town, Cape Agulhas (most southern point in Africa) and neighbouring major river systems.

*Pseudobarbus verloreni* is a lowland species that is restricted to the Verlorenvlei River system on the west coast of South Africa (Figure 5). The morphological features of two juvenile specimens of *Pseudobarbus* collected from the Langvlei River by Thorne and Cambray in 1986 (SAIAB 130464) are consistent with juveniles of the new species, and are thus assigned to *Pseudobarbus verloreni*. The Langvlei River population is likely to have been extirpated, as no specimens of *Pseudobarbus* have been collected during more recent surveys (2001–2012). The major impact on this river is excessive water extraction that causes the river to dry up completely during the dry season. The Verlorenvlei River system has a gentle gradient and slow to moderate flow for much of the year. The water is highly turbid during the rain season (winter months) when water volume and flow velocity is high, but it becomes less turbid during low flow periods. Most sections of the river system recede into a series of isolated pools during the dry season, especially during late summer and autumn. The bottom substratum is predominantly sand, silt and mud. This is in contrast with the majority of the streams in the CFR that are associated with the Cape Fold Mountains with steeper gradients, clear water, moderate to fast flow throughout the year and rocky substratum. The species was possibly widespread throughout the Verlorenvlei and Langvlei River systems in the past, but numbers likely declined during the last century due to predation and competition from introduced fish species and habitat degradation (see below).

#### Etymology.

The species is named after the Verlorenvlei River system to which it is now confined.

#### Conservation.

The Verlorenvlei redfin was listed as Endangered during the most recent IUCN assessment by Tweddle et al. (2009). The presence of non-native predatory black bass *Micropterus* spp and potential competitors, banded tilapia *Tilapia sparrmanii*, Mozambique tilapia *Oreochromis mossambicus* and common carp *Cyprinus carpio*, habitat degradation and excessive water withdrawal for agricultural purposes pose the greatest threat to the survival of this species. Protection of critical habitats and establishment of sanctuaries are some of the most immediate conservation measures required to prevent further decline. The effectiveness of current protected areas in conserving *Pseudobarbus verloreni* is limited because they largely encompass upland areas where this species does not occur. Long-term measures to protect and prevent extinction of this species may have to include eradication of alien fishes and the construction of barriers to prevent re-invasion where feasible and restoration of existing habitats to facilitate recovery.

### Key to double barbeled redfin species of the genus *Pseudobarbus*

**Table:** 

1	Mouth terminal, 36–39 lateral scale series	***Pseudobarbus skeltoni***
–	Mouth sub-terminal	**2**
2	Lower lip unretractable, cartilaginous fig absent, conspicuous linear speckles above and below lateral line	***Pseudobarbus verloreni* sp. n.**
–	Lower lip retractable, cartilaginous fig present	**3**
3	Anterior barbels less than 30% of orbit diameter	***Pseudobarbus burgi***
–	Anterior barbels more than 30% of orbit diameter	***Pseudobarbus burchelli***

## Discussion

Specimens of *Pseudobarbus* from the Verlorenvlei River system show clear genetic and morphological differences when compared with the three currently described double barbeled *Pseudobarbus* species (*Pseudobarbus burchelli*, *Pseudobarbus burgi*, and *Pseudobarbus skeltoni*) and are thus described as a new species. The morphological differentiation between *Pseudobarbus verloreni* sp. n. and *Pseudobarbus burgi* reported here is consistent with the findings of Skelton (1988) who reported considerable ‘intraspecific’ morphological variation between Verlorenvlei and Berg populations. The most informative characters that distinguish *Pseudobarbus verloreni* sp. n. from *Pseudobarbus burgi* are body depth, head length, predorsal length, snout length and anterior barbel length. However, *Pseudobarbus verloreni* sp. n. and *Pseudobarbus burgi* cannot be distinguished based on meristic characters because considerable overlap exists between the two species.

Phylogenetic results from the present study, Swartz et al. (2009) and Chakona and Swartz (2013) show that the relationships among *Pseudobarbus verloreni* sp. n. (referred to as Verlorenvlei lineage in latter two studies), *Pseudobarbus burgi* sensu stricto, *Pseudobarbus burchelli* sensu lato and *Pseudobarbus skeltoni* are not well resolved, with a polytomy linking the new species and the other taxa. This is further evidence that the *Pseudobarbus* from Verlorenvlei represents a separate species as it does not clearly group with one of the other species or lineages. Our review of available material of double barbeled redfins confirmed that *Pseudobarbus verloreni* sp. n. is restricted to the Verlorenvlei River system and likely have been extirpated from the adjacent Langvlei River system.

Reduced tubercle occurrence in *Pseudobarbus verloreni* could represent a different breeding strategy or behaviour compared to other redfins. Further research is required to better describe the ecology, biology, population size, distribution and conservation status of this species. There are serious conservation concerns, because this species is associated with pool habitats, which are also favourable habitats for non-native fish predators and competitors. This species is also threatened by proposed mining activities and excessive water withdrawal in the Verlorenvlei catchment. Improved understanding of the conservation status, distribution and ecology is a critical requirement for developing effective conservation measures to prevent extinction of this species. The current protected areas are unlikely to be effective for the conservation of *Pseudobarbus verloreni* as the known distribution range of this species falls outside protected areas. Expansion of protected areas and education of landowners may be necessary to ensure survival of this species.

## Supplementary Material

XML Treatment for Pseudobarbus verloreni
